# Rotavirus Inner Capsid VP6 Acts as an Adjuvant in Formulations with Particulate Antigens Only

**DOI:** 10.3390/vaccines8030365

**Published:** 2020-07-07

**Authors:** Suvi Heinimäki, Kirsi Tamminen, Vesa P. Hytönen, Maria Malm, Vesna Blazevic

**Affiliations:** 1 Vaccine Development and Immunology/Vaccine Research Center, Faculty of Medicine and Health Technology, Tampere University, 33520 Tampere, Finland; suvi.heinimaki@tuni.fi (S.H.); kirsi.tamminen@tuni.fi (K.T.); maria.malm2@gmail.com (M.M.); 2 Protein Dynamics Group, Faculty of Medicine and Health Technology, Tampere University, 33520 Tampere, Finland; vesa.hytonen@tuni.fi; 3 Fimlab Laboratories, Pirkanmaa Hospital District, 33520 Tampere, Finland

**Keywords:** adjuvant, alum, VP6, nanostructure, P particle, peptide, M2e

## Abstract

Novel adjuvants present a concern for adverse effects, generating a need for alternatives. Rotavirus inner capsid VP6 protein could be considered a potential candidate, due to its ability to self-assemble into highly immunogenic nanospheres and nanotubes. These nanostructures exhibit immunostimulatory properties, which resemble those of traditional adjuvants, promoting the uptake and immunogenicity of the co-administered antigens. We have previously elucidated an adjuvant effect of VP6 on co-delivered norovirus and coxsackievirus B1 virus-like particles, increasing humoral and cellular responses and sparing the dose of co-delivered antigens. This study explored an immunostimulatory effect of VP6 nanospheres on smaller antigens, P particles formed by protruding domain of a norovirus capsid protein and a short peptide, extracellular matrix protein (M2e) of influenza A virus. VP6 exhibited a notable improving impact on immune responses induced by P particles in immunized mice, including systemic and mucosal antibody and T cell responses. The adjuvant effect of VP6 nanospheres was comparable to the effect of alum, except for induction of superior mucosal and T cell responses when P particles were co-administered with VP6. However, unlike alum, VP6 did not influence M2e-specific immune responses, suggesting that the adjuvant effect of VP6 is dependent on the particulate nature of the co-administered antigen.

## 1. Introduction

Inner capsid VP6 protein of the triple-layered rotavirus (RV) particle exhibits polymorphic nature, being able to spontaneously assemble in vitro into trimers and further diverse and highly immunogenic nanostructures, including nanospheres and nanotubes. The type of VP6 assembly can be affected mainly by altering pH, ionic strength and divalent cation concentration [1,2]. The highly organized multivalent antigen expression, particulate nature, size and morphology [3,4,5] make these VP6 assemblies strong inducers of humoral and cellular immune responses through the efficient cross-linking of B cell receptors [6] and internalization by antigen-presenting cells (APCs) [7,8].

Due to the high conservation and immunogenicity of VP6 protein, subunit vaccine candidates against RV based on VP6 have been considered a safe alternative to the current live attenuated oral RV vaccines [9,10]. We have developed a combination vaccine candidate against childhood gastroenteritis containing RV VP6 nanostructures and norovirus (NoV) virus-like particles (VLPs) [11,12], formed by self-assembly of major capsid protein VP1 [13]. Based on comprehensive preclinical studies, the candidate NoV-RV combination vaccine is highly immunogenic, inducing protective immune responses against both viruses [11,12,14]. During development of the combination vaccine, VP6 nanotubes and nanospheres were discovered to function also as an immunomodulator and delivery vehicle for co-administered NoV VLPs, promoting the uptake and immunogenicity as well as reducing the dose of the co-administrated antigens [15,16]. In addition, we recently elucidated that adjuvant effect of VP6 nanoparticles is not restricted to NoV VLPs, but they can improve immune responses to coxsackievirus B1 (CVB1) VLPs as well [17]. In addition to our studies, others have shown that VP6 nanospheres enhance responses to short heterologous peptides when employed as carriers [18,19,20].

Adjuvants are defined as compounds or molecules with intrinsic immunomodulatory properties, which effectively potentiate immune responses to vaccine antigens. Currently, only a few adjuvants (alum, oil-in-water emulsions, virosomes and adjuvant system combinations such as AS01, AS03, AS04) have been approved for clinical use, insoluble aluminum salts (alum) being the first and widely used adjuvant included in the formulation of many licensed human vaccines. While alum is employed mainly to adsorb the vaccine antigen and as a delivery system [21,22], it has also been described to have some immunomodulatory features [23]. However, because of the local and systemic adverse effects associated with adjuvanted vaccines [24], alternatives to current and potentially harmful adjuvants are being considered.

Due to the observed immunostimulatory action on co-delivered 30–40 nm VLPs, VP6 might be considered as a potential adjuvant to improve immunogenicity of different vaccine antigens. Thus, the present study was aimed to investigate, whether VP6 nanospheres also act as an adjuvant for smaller antigens with apparently lower immunogenicity by selecting two model antigens, ~20 nm NoV P particle (the estimated molecular mass of 830 kDa) formed by 12 dimers of a specific exterior protruding (P) domain (34.5 kDa) of the NoV VP1 capsid [25,26] and a 23-mer extracellular domain of matrix protein M2 (M2e, a monomeric peptide, ~3 kDa) of influenza A virus. The current study reveals that VP6 potentiated antibody and T cell immune responses against P particles, but not against a M2e peptide, suggesting that VP6 nanostructures are able to promote immunogenicity of particulate antigens only.

## 2. Materials and Methods

### 2.1. Antigenic Formulations

#### 2.1.1. Antigen Production and Characterization

NoV GII.4 P particles (1999, acc. no. AF080551) were expressed in *E. coli* (BL21 star) cells and purified by Ni-NTA affinity chromatography according to the previously published procedures [27]. RV VP6 (acc. no. GQ477131) nanospheres were produced by recombinant baculovirus (BV) technology in Sf9 cells and purified on sucrose gradients and ultracentrifugation [11], followed by ultrafiltration procedures [28].

The purity, identity, and morphology of the P particles and VP6 nanospheres were characterized according to previously described procedures [28]. These included confirmations of BV absence by a BacPAK Rapid Titer kit (Takara Bio Inc, Kusatsu, Japan) and SDS-PAGE followed by immunoblotting with anti-BV gp64 antibody (Santa Cruz Biotechnology Inc, Santa Cruz, CA, USA) as well as determination of endotoxin levels using ToxinSensorTM Gel Clot Endotoxin Assay kit (GenScript, Piscataway, NJ, USA). The integrity and morphology were examined by transmission electron microscopy (TEM) with Jeol JEM-1400 (Jeol Ltd., Tokyo, Japan). Furthermore, hydrodynamic diameter, volume distribution and polydispersity index (PdI) were determined by dynamic light scattering (DLS) with a Zetasizer Nano ZS (Malvern Instruments Ltd., Worcestershire, UK).

For immunological assays, five different NoV capsid VLPs derived from GII.4 (1999, acc. no. AF080551), GII.4 New Orleans (NO; 2010, acc. no. GU445325), GII.4 Sydney (Syd; 2012, acc. no. AFV08795.1), GII.12 (1998, acc. no. AJ277618), and GII.17 (2015, acc. no. BAR42289) were produced in BV-insect cell expression system and purified by sucrose gradient ultracentrifugation as described in detail elsewhere [11].

#### 2.1.2. Synthetic Peptides

Two 23-mer peptides derived from M2e protein of human (SLLTEVETPIRNEWGCRCNDSSD) and swine (SLLTEVETPTRSEWECRCSDSSD) influenza A virus were synthetized by Synpeptide Co. Ltd. (Shanghai, China). The lyophilized M2e peptides were dissolved at 30 mg/mL in DMSO (Sigma-Aldrich, St Louis, MO, USA).

### 2.2. Mice Immunization

Pathogen-free female 6-week-old BALB/c OlaHsd mice (Envigo, Horst, the Netherlands) were randomly divided into seven groups (Gr I-VII, 4–5 mice/group), and acclimatized under controlled specific conditions for a period of one week before starting the experiment. Animals were immunized twice with subcutaneous (s.c.) injection into the right flank with P particles or M2e peptide (human sequence) alone, in combination with VP6 or formulated with Al(OH)_3_ (Alhydrogel; InvivoGen, San Diego, CA, USA) at a 3-week interval. Table 1 depicts the employed antigenic formulations, each diluted in sterile PBS (Lonza, Verviers, Belgium) to contain the indicated dose of immunogen. The control group received no antigen. Immunizations were conducted under general anesthesia by inhalation of isoflurane (Attane vet, Vet Medic Animal Health Oy, Parola, Finland).

Tail blood samples were drawn from each mouse prior to the immunizations (at study weeks 0 and 3) to monitor the development of the antibody responses. The mice were euthanized on study week 5 and sera, fecal samples and splenocytes were collected as previously described [11]. The preclinical study was performed under the regulations and guidelines of the Finnish National Experiment Board (Permission number ESAVI/10800/04.10.07/2016). The welfare of the animals was monitored during the study and every effort was made to minimize animal suffering.

### 2.3. Antigen–Specific Antibody Responses

Antibody responses generated against NoV GII.4 P particles and RV VP6 nanospheres were determined measuring NoV GII.4 type-specific and cross-reactive as well as RV VP6-specific IgG antibody levels in serum samples of individual mice by ELISA. Furthermore, 10% fecal suspensions were studied for anti-GII.4 IgG antibodies. The procedural steps of the employed ELISA were similar to those of previously published work [11], and are only shortly outlined as follows: Half-area polystyrene plates (Corning Inc., Corning, NY, USA) were coated with 50 ng of NoV P particles or VLPs (GII.4, GII.4 NO, GII.4 Sydney, GII.12, and GII.17) or RV VP6 per well. Antigen-specific antibodies in serum and fecal specimens were detected with a combination of horseradish peroxidase-conjugated anti-mouse IgG (Sigma-Aldrich, St Louis, MO, USA), IgG1 (Invitrogen, Carlsbad, CA, USA) or IgG2a (Invitrogen) and SIGMA FAST OPD substrate (Sigma-Aldrich). Optical densities at 490 nm (OD_490_) were measured by Victor2 microplate reader (PerkinElmer, Waltham, MA, USA). Endpoint titers were expressed as the reciprocal of the highest sample dilution with an OD_490_ above the cut-off value (>0.1 OD_490_ unit).

Induction of antibody responses by M2e vaccine formulations was determined measuring type-specific and cross-reactive anti-M2e IgG antibodies in the sera of individual mice as described above for NoV GII.4- and RV VP6-specific responses, but the microtiter plates were coated with the M2e peptides derived from human or swine influenza A virus (500 ng/well) and detection of antibodies was accomplished with TMB substrate kit (Vector Laboratories, Burlingame, CA, USA). The OD readings were measured at 450 nm (OD_450_). A titer of 50 was assigned to negative samples, being a half of the starting serum dilution.

### 2.4. Avidity of NoV Antibodies

The avidity of NoV GII.4-specific IgG antibodies was determined using urea-based ELISA assay in which high-concentration urea is used to elute off the low-avidity antibodies [29,30]. The assay was performed according to the ELISA method described above for GII.4 VLPs, but after the serum (diluted 1:200) incubation step, an additional urea treatment was performed to eliminate the antibodies with low-avidity. Results were expressed as avidity index: (OD_490_ with urea/OD_490_ without urea) × 100%.

### 2.5. NoV Blocking Assay

Blocking assay, a surrogate neutralization assay for NoV, was used to measure the ability of the antibodies to inhibit the binding of NoV VLPs to cellular binging ligands, histo-blood group antigens (HBGA), employing pig gastric mucin (PGM) type III (Sigma Chemicals, St Louis, MO) as the source of HBGAs [17,31]. Briefly, GII.4 VLPs were preincubated with serially diluted sera (starting from 1:100 dilution), after which the mixtures were added on PGM coated microwell plates, followed by the detection of the bound VLPs with human GII.4 antiserum and anti-human HRP-conjugated secondary IgG (Novex; Thermo Fisher Scientific, Fremont, CA, USA). The blocking indexes were calculated for each serum dilution with the following formula: 100%-[(OD_490_ sample/OD_490_ max. binding) × 100%]. The fifty-percent blocking titer (BT_50_) was determined as the reciprocal of the final serum dilution blocking ≥50% of the maximum VLP binding defined with VLPs lacking serum pre-incubation. Sera failing to block at least 50% of the binding were assigned with a BT_50_ of 50 (half of the starting dilution).

### 2.6. NoV-Specific ELISPOT IFN-γ

Activation of T cell immunity by P particle formulations was analyzed by quantification of IFN-γ production from splenocytes of individual mice using an ELISPOT-IFN-γ assay [12] where bone marrow-derived dendritic cells (BMDCs) pulsed with NoV GII.4 VLPs were employed as APCs as we recently described [16]. For detection of IFN-γ producing cells, the liquid nitrogen frozen splenocytes (0.2 × 10^6^ cells/well) were stimulated in duplicates with autologous BMDCs (20,000 BMDCs/well) previously pulsed with NoV VLPs or left unpulsed. Culture medium was used as background control and the viability of the splenocytes was controlled by Concanavalin A (T cell mitogen, Sigma-Aldrich) stimulation. The spots representing individual IFN-γ secreting cells were automatically calculated by ImmunoSpot^®^ CTL analyzer (CTL-Europe, GmbH, Bonn, Germany). The results were expressed as mean spot-forming cells (SFC)/10^6^ splenocytes.

### 2.7. Statistical Analyses

Mann–Whitney U-test and Kruskal–Wallis tests were used to calculate the statistical differences between the non-parametric observations. The data were analyzed with GraphPad Prism software (San Diego, CA, USA), version 8.0.1, and *p* < 0.05 was defined to indicate the statistically significant differences.

## 3. Results

### 3.1. Characterization of Vaccine Antigens

**Figure 1:**
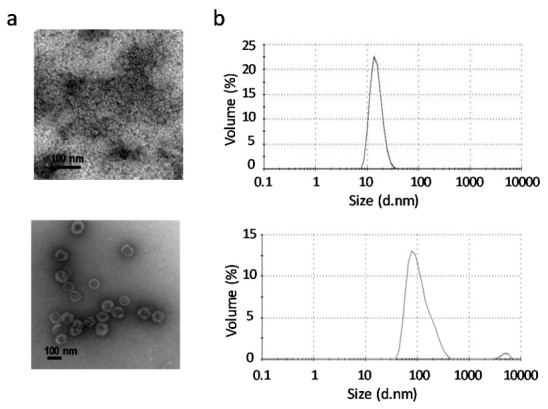
Characterization of the vaccine antigens. (**a**) Electron micrographs of norovirus (NoV) P particles (left panel) and rotavirus (RV) VP6 nanospheres (right panel). Black bar indicates 100 nm. (**b**) Dynamic light scattering (DLS) analysis of the vaccine antigens. Shown are size distributions in nanometers (nm) by volume percent of NoV P particles (left panel) and RV VP6 nanospheres (right panel). The results are presented as the average of 3–5 measurements (each measurement containing 10–20 × 10 s datasets at 25 °C).

Purity, integrity and morphology of GII.4 P particles and VP6 protein were confirmed as described in Materials and Methods. No residual impurities were observed in the purified products, as antigens were free of bacterial endotoxins (<0.02 EU/10 µg protein) [32], live BV (0 pfu/mL) as well as BV gp64 protein. Both antigen preparations consisted of particles of the expected size and morphology, including GII.4 P particles or VP6 nanospheres, confirmed under TEM (Figure 1a).

The size distribution profiles of the antigenic formulations analyzed by DLS showed that 100% of the GII.4 P particles and VP6 nanospheres resulted in the respective average hydrodynamic diameters of 15.33 ± 3.99 nm (PdI 0.026) and 115.6 ± 60.99 nm (PdI 0.181) (Figure 1b), which are concurrent with the sizes determined by TEM.

### 3.2. VP6 Effect on Immunogenicity of P Particles

#### 3.2.1. Induction of Robust Anti-GII.4 Antibody Response

**Figure 2:**
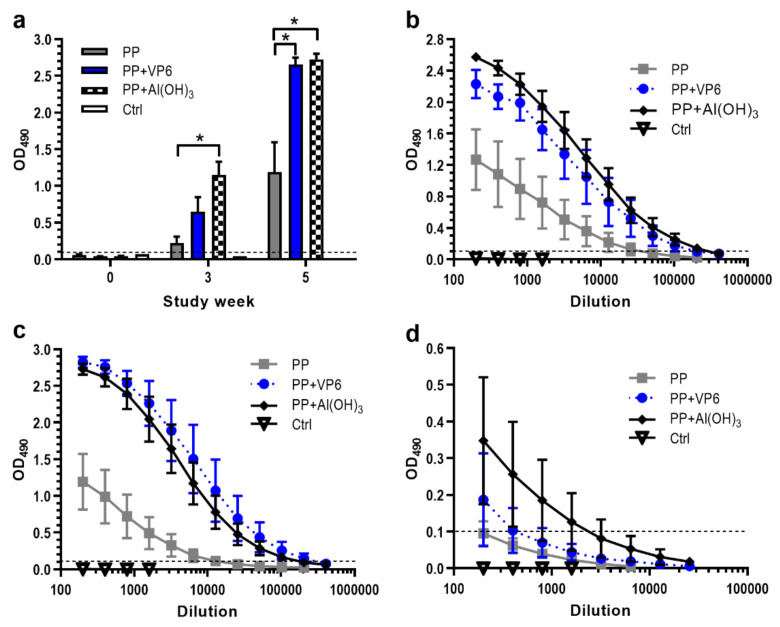
Development of serum antibody responses against norovirus P particles (PP) following immunization with PP alone or formulated with VP6 or Al(OH)_3_. Control (Ctrl) received no immunogen. (**a**) Kinetics of total IgG antibodies following immunizations at study weeks 0 and 3. Group mean OD_490_ values with standard errors of the means of 1:200 diluted sera at indicated study weeks are shown. * indicates statistically significant (*p* > 0.05) differences between the groups. Endpoint titration curves of serum IgG (**b**) IgG1 (**c**) and IgG2a (**d**) antibodies in termination sera at study week 5. Shown are mean titration curves with standard errors of the means of the experimental groups. Horizontal dashed lines indicate the cut-off level (OD_490_ ≥ 0.1).

To examine the effect of VP6 nanospheres on the immunogenicity of NoV GII.4 P particles, mice were immunized twice with P particles alone or in combination with VP6. For comparison, one experimental group received P particles formulated with Al(OH)_3_. Figure 2a depicts the development of serum IgG antibodies against P particles at study weeks 0, 3 and 5. Three weeks after the first dose, IgG was present in all immunized mice. However, the first dose of P particles alone induced only minor IgG response (OD_490_ 0.223 ± 0.085), antibody levels being three- or five-fold lower than the levels induced by co-administration of P particles with VP6 (*p* = 0.063) or Al(OH)_3_ (*p* = 0.008). The second doses of each formulation, administered at week 3, further enhanced GII.4-specific responses, as observed at week 5. P particles and VP6 or Al(OH)_3_ formulations led to the levels that were significantly higher (*p* = 0.015) than those seen with P particles alone. The IgG titer after immunization with P particles and VP6 ranged from 4.4 to 5.3 log_10_, with a geometric mean titer (GMT) of 4.9 log_10_ (Figure 2b). This was nine-fold higher level elicited by P particles alone (GMT 4 log_10_), but the same approximate level elicited by P particles formulated with Al(OH)_3_ (GMT 5.1 log_10_).

Determination of GII.4-specific IgG subtype IgG1 and IgG2a titers, representing Th2- and Th1-type responses, revealed the induction of IgG1 antibodies with each P particle formulation (Figure 2c). Co-administration of P particles with VP6 (GMT 5.2 log_10_) or Al(OH)_3_ (GMT 5 log_10_) generated 39 or 27-fold greater levels of IgG1 (*p* = 0.014) compared to administration of P particles alone (GMT 3.6 log_10_) (Figure 2c). Although the inclusion of VP6 or Al(OH)_3_ in the PP formulation increased IgG2a antibody magnitudes slightly (Figure 2d), the differences were statistically insignificant (*p* = 0.383 or *p* = 0.156). Control mice remained negative for anti-GII.4 antibodies during the whole study period (Figure 2a–d).

#### 3.2.2. Effect of VP6 on Functionality of NoV-Specific Antibodies

**Figure 3:**
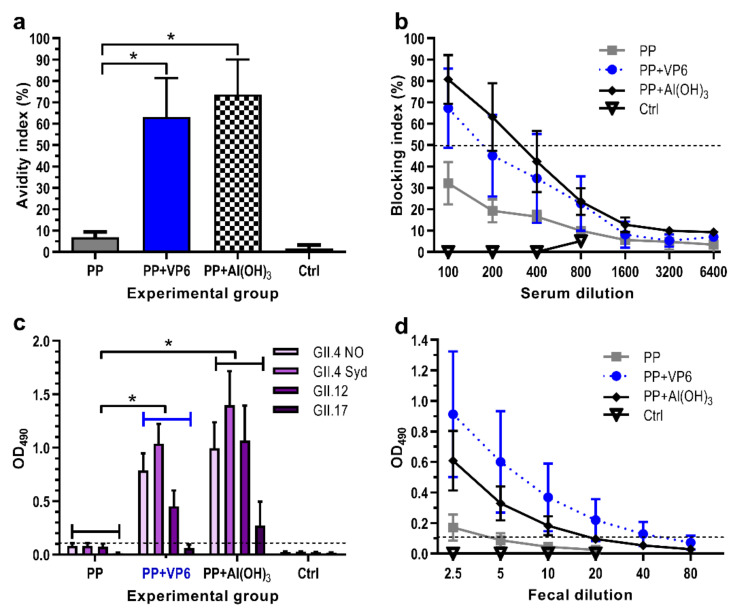
Functionality of antibodies following immunization with norovirus (NoV) P particles (PP) alone or formulated with VP6 or Al(OH)_3_. Control group (Ctrl) received no immunogen. (**a**) Avidity of serum IgG antibodies against NoV GII.4 VLPs. Mean avidity indices (%) with standard errors of the means of the groups are shown. (**b**) Homologous blockage of GII.4 VLP binding to HBGA by serum antibodies. Shown are the mean blocking indices (%) with standard errors of the means of the experimental groups. The horizontal dashed line represents the blocking titer 50% (BT_50_). (**c**) Cross-reactive serum IgG responses against heterologous NoV VLPs. Group mean OD_490_ values with standard errors of the means of 1:200 diluted sera are shown. Horizontal dashed line indicates the cut-off level (OD_490_ ≥ 0.1). (**d**) Endpoint titrations of fecal IgG antibodies against NoV GII.4 VLPs. Shown are mean titration curves with standard errors of the means of the experimental groups. Horizontal dashed line indicates the cut-off level (OD_490_ ≥ 0.1). * indicates statistically significant (*p* > 0.05) differences between the groups.

Immune sera from each individual mouse were assayed for the avidity of anti-GII.4 IgG antibodies. Immunization with P particles alone induced IgG antibodies with extremely low avidity (avidity index 6.9 ± 2.5%), but addition of VP6 or Al(OH)_3_ in the formulation elevated significantly (*p* = 0.016 for both) the avidity of antibodies (respective avidity indices 63.2 ± 18.2% and 73.7 ± 16.4%) (Figure 3a).

Ability of induced NoV-specific antibodies to block binding of GII.4 VLPs to the HBGAs was examined as a protective potential of antisera. All experimental groups receiving P particle formulations developed antibodies with detectable blocking activity (Figure 3b). However, the co-administration of P particles with VP6 or Al(OH)_3_ resulted in 2–3-fold rise (*p* = 0.043) in the GMTs of BT_50_ (BT_50_ 141 or 174, respectively) than administration of P particles alone (BT_50_ 57).

The functionality was further examined measuring the cross-reactivity of the serum antibodies against four heterologous NoV VLPs derived from genogroup II, including GII.4 NO, GII.4 Sydney, GII.12, and GII.17. No cross-reactive IgG antibodies were detected after immunization with P particles alone, while co-administration with VP6 or Al(OH)_3_ resulted in antibodies with significantly broader (*p* < 0.001) cross-reactivity (Figure 3c). Overall, the weakest responses were induced against GII.17 VLPs.

To assess the adjuvant effect of VP6 on generation of antibodies at mucosal surfaces, individual 10% fecal suspensions of each immunized mouse were tested for the presence of anti-GII.4 IgG. Immunization with P particles alone induced extremely low levels of fecal antibodies (GMT 2.5), but the magnitudes of intestinal antibodies were increased by formulation of the antigen with VP6 (GMT 20, *p* = 0.035) or Al(OH)_3_ (GMT 8.7, *p* = 0.11) (Figure 3d). No antigen-specific mucosal responses were detected in the control mice (Figure 3d).

#### 3.2.3. Increase in GII.4-Specific T Cell Responses by VP6

**Figure 4:**
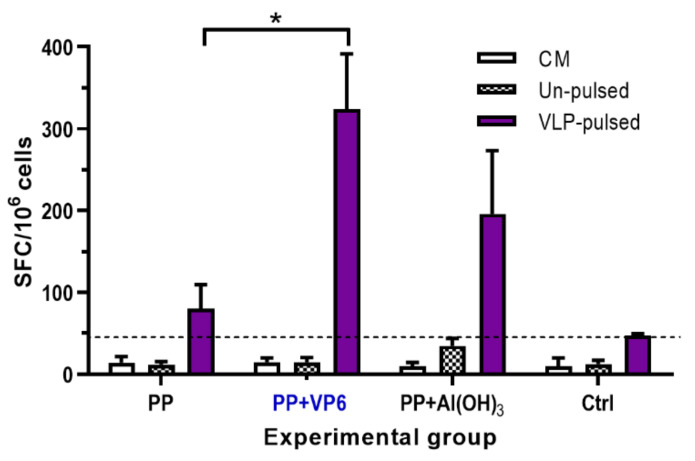
T cell responses following immunization with norovirus (NoV) P particles (PP) alone or formulated with VP6 or Al(OH)_3_. Control (Ctrl) received no immunogen. Shown are IFN-γ responses following stimulation of splenocytes with un-pulsed or NoV GII.4 VLP -pulsed BMDCs. Results are expressed as mean IFN-γ spot forming cells (SFC)/10^6^ cells with standard error of the means of the experimental groups. The dashed line indicates the cut-off limit obtained from cells incubated in a culture medium (CM) only (mean SFC/10^6^ + 3 × SD). * indicates statistically significant (*p* > 0.05) differences between the groups.

The induction of T cell responses by P particle formulations was characterized analyzing Th1-type cytokine IFN-γ production from the splenocytes of individual mice. Cells from the mice receiving P particles alone did not produce considerable levels of IFN-γ in response to VLP-pulsed BMDCs, but appreciable high quantities of IFN-γ secreting cells were elicited by the co-administration of P particles with VP6 (*p* = 0.014) or Al(OH)_3_ (*p* = 0.075) (Figure 4). Despite the superior T cell responses induced by P particles and VP6, no statistically significant difference was detected between VP6 or Al(OH)_3_ (*p* = 0.085). Irrespective of vaccine formulation, splenocytes from experimental mice did not respond with IFN-γ release, when stimulated with un-pulsed BMDCs (Figure 4). No T cell responses were developed by the cells of negative control mice (Figure 4). Culture media alone stimulated no IFN-γ secretion by the cells from any of the groups (Figure 4).

### 3.3. Induction of M2e-Specific Serum Antibodies by Al(OH)_3_ Only

**Figure 5:**
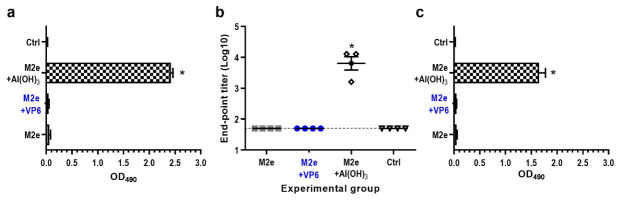
Development of serum antibody responses against M2e following two immunizations with M2e alone or in combination with VP6 or Al(OH)_3_. Control (Ctrl) received no immunogen. (**a**) Group mean OD_450_ values with standard errors of the means of IgG antibodies in 1:100 diluted termination sera. (**b**) Endpoint titers of serum IgG antibodies in termination sera. Each symbol represents an individual mouse. The solid line indicates the geometric mean titer of the group. A titer of 50 (1.7 log_10_) was assigned for sera with no detectable antibodies, being a half of the initial serum dilution. Horizontal dashed line indicates the cut-off level (1.7 log_10_). (**c**) Cross-reactive serum IgG responses against heterologous M2e peptide derived from swine influenza virus. Shown are group mean OD_490_ values with standard errors of the means of IgG antibodies in 1:100 diluted termination sera. * indicates statistically significant (*p* > 0.05) differences between the groups.

The adjuvant effect of VP6 on antibody responses induced by the peptide was investigated immunizing mice with a 23-mer M2e peptide of human influenza A virus. Immunization with M2e peptide alone or in combination with VP6 did not induce serum IgG antibodies, whereas the coadministration of M2e peptide with Al(OH)_3_ resulted in significant (*p* = 0.002) M2e-specific IgG levels (GMT 3.8 log_10_) (Figure 5a,b). Similarly, only formulation of M2e peptide with Al(OH)_3_ generated cross-reactive antibody response against M2e peptide derived from swine influenza A virus (Figure 5c).

### 3.4. Development of VP6-Specific Serum Antibody Responses

In order to confirm the success of the VP6 administration, serum anti-VP6 IgG levels were analyzed. Each mouse receiving 10 µg of VP6 nanospheres in combination with P particles or M2e developed robust IgG response to VP6 (GMT 4.4 log_10_) (data not shown). None of the mice in the control or other experimental groups developed VP6-specfic antibodies (data not shown).

## 4. Discussion

A challenge in development of subunit vaccines is the poor immunogenicity of some vaccine antigens, including soluble monomeric proteins and short peptides, unless administered with a powerful adjuvant. Due to the potential toxicity and adverse effects of adjuvants, such as elevated levels of pro-inflammatory mediators, enhanced reactogenicity and the breakdown of self-tolerance [24], there is a need for alternative strategies. Our previous studies have demonstrated VP6 nanotubes and nanospheres to possess pronounced adjuvant characteristics, promoting adaptive immune responses against co-administered antigens and the activation of innate immunity [15,17], independently from the type of the nanostructures. VP6 nanospheres have also been reported by others to improve the responses to the foreign peptides or proteins, when exploited as immunological carriers [18,19,20]. Hence, the potent immunostimulatory and immunomodulatory properties of VP6 nanostructures make them conceivable candidates to be considered for the improvement of immunological responses to various vaccine antigens. Based on former adjuvant action of VP6 on co-delivered NoV GII.4 and GI.3 as well as CVB1 VLPs [12,17], the present study was extended to explore the influence of VP6 on immunogenicity of two different model antigens—NoV GII.4 P particle (24-mer of ~830 kDa, ~20 nm in diameter) and a short M2e peptide (23 aa, ~3 kDa and an estimated diameter ~1.5 nm). VP6 function was compared to that of alum, which is considered a gold standard delivery or depot system and the adjuvant of choice for injectable vaccines.

Congruent with our previous findings with NoV VLPs, we detected the superior immunogenicity of P particles, when formulated with VP6 nanospheres. The observed immunostimulatory effect of VP6 was comparable to the effect of alum. Inclusion of VP6 in the formulation contributed not only to the induction of P particle-specific antibodies, but also the functionally efficient responses against NoV VLPs, as VP6 nanospheres considerably increased the avidity of serum IgG antibodies as well as the blocking potential of the induced antibodies. Antibodies with high avidity have been demonstrated to contribute to virus neutralization [29,33] and protection from some viral infections [34,35]. Furthermore, antibodies which prevent the binding of NoV VLPs to the HBGAs, cellular attachment factors or putative receptors for NoV [36,37,38], are considered the best correlate of protection against NoV infection [36,39]. Despite the lack of the conserved interior shell domain of capsid VP1 in P particles [25], VP6 also stimulated the induction of cross-reactive antibodies towards heterologous NoV genotypes, which is essential to provide cross-protection against a number of existing and future evolving genotypes of NoVs [40].

Although VP6 nanospheres seemed to skew antibody isotype distribution of the antibody response towards the IgG1 subtype, functioning preferably as a Th2 adjuvant like alum, VP6 can also be considered a potent Th1-type adjuvant. VP6 promoted the generation of T cell responses against co-delivered P particles, as addressed by the substantial secretion of IFN-γ cytokine, a hallmark of Th1 cell immunity, by antigen-primed splenocytes upon stimulation with NoV VLP-pulsed BMDCs. Due to the gut mucosa as the port of entry for enteric viruses and possible contribution of T cell immunity in the generation of heterologous immunity [41,42], induction of the stronger mucosal response and cell-mediated immunity by VP6 is of a high relevance in NoV infection.

Small antigens require an adjuvant for stimulation of strong immune responses [43]. Although VP6 nanospheres exhibited a pronounced adjuvant effect on B and T cell responses induced by NoV P particles, no adjuvant effect of VP6 on M2e peptide was detected. Instead, the determination of anti-M2e IgG revealed the induction of humoral immunity only by M2e formulated with alum. As very high levels of VP6-specific IgG were induced in the mice immunized with a combination of M2e and VP6, the lack of VP6 adjuvant effect due to unsuccessful immunization with VP6 can be ruled out. Consistent with this observation, we also detected the absence of the immunostimulatory action of VP6 on ovalbumin, a soluble monomeric protein (unpublished observation). These data suggest that VP6 nanostructures can only act as an adjuvant for particulate multimeric antigens but not for soluble monomeric antigens or short peptides. In addition, the size of the particles (VLPs and P particles, respectively) and their three-dimensional structure closely resembling viruses is important.

For the induction of potent immune responses, an antigen needs to traffic efficiently from the injection site to secondary lymphoid organs such as lymph nodes or spleen for activation of T and B cells [43]. The observed differences in VP6 adjuvanticity for particulate versus soluble monomeric proteins and peptide antigens may originate (1) from different antigen persistence at the injection site, (2) different uptake by APCs and (3) different biodistribution of peptides and soluble antigens compared to P particles and VP6 nanospheres. The trafficking to the draining lymph nodes depends on the size and the particulate nature of the antigen, ~20–200 nm particles preferentially draining rapidly through lymphatic vessels, and larger ones trafficking more slowly in a DC-dependent manner [4,5,43,44]. Instead, very small (<5 nm) antigens disseminate into the systemic circulation with poorer lymphatic drainage and rapid release, contributing to the low immunogenicity of peptides and other soluble antigens [44,45]. Therefore, it seems likely that similar size and trafficking of VP6 nanospheres and P particles has an impact on the adjuvant effect observed for VP6.

Alum has been originally described as a good adsorbent through the formation of a depot, providing prolonged antigen exposure and enhanced antigen release, uptake and stability at the injection site [21,24,46]. In comparison, VP6 may function as a carrier for co-administered particulate antigens by forming aggregates at the injection site, which could explain the spatial and temporal requirement for co-delivery and co-localization of antigens. The adjuvant action of VP6 probably derives from the capture of the nanostructures by macrophages and DCs, which triggers the APC activation and maturation [15,16] regardless of the structural assembly [47]. This, in turn, leads to the upregulation of antigen presentation and co-stimulatory molecules, as well as the secretion of pro-inflammatory cytokines [15], recruiting other APCs at the injection site, modulating conditions promoting uptake and presentation of co-delivered antigens and facilitating migration of APCs to lymph nodes [4]. On the other hand, we have recently indicated that co-administered NoV VLPs can enter the same cells with VP6 [16], which suggests the travel of co-administered antigens together to the lymphoid tissue.

In conclusion, the present study demonstrates that the presence of VP6 in P particle formulation increased both antibody and T cell immune responses. The observed effect of VP6 was comparable to that of alum, apart from the higher mucosal and T cell responses raised by VP6. Thus, VP6 not only acts as an adjuvant for VLPs, but also for smaller particles. However, we observed no adjuvant effect of VP6 on M2e peptide-induced immunity, suggesting the stimulatory action to be dependent on the nature and characteristics of the employed vaccine antigen. Therefore, these data support the notion that VP6 nanostructures could function as a potent adjuvant for particulate antigens, especially when cell-mediated immune responses are essential for protection.

## 5. Conclusions

This study explored an adjuvant effect of VP6 nanospheres on immunogenicity of two model antigens, P particles formed by protruding domain of a norovirus capsid VP1 protein and a short peptide derived from a matrix protein M2e of influenza A virus. The results demonstrated that VP6 exhibited a considerable improvement of immune responses induced by P particles. Due to the lack of adjuvant effect on M2e peptide-specific responses, the adjuvant action of VP6 nanospheres appears to be dependent on the particulate nature and size of the co-administered antigen. Thus, VP6 could be considered as an alternative to current and potentially harmful adjuvants for improvement of antibodies and T cell responses to particulate vaccine antigens.

## Figures and Tables

**Table 1:** Antigenic formulations and immunization of experimental mice.

Experimental Group	Immunogen	Injection Dose (µg)	# Mice/Group
I	PP	10	5
II	PP + VP6	10 + 10	4
III	PP + Al(OH)_3_	10 + 100	5
IV	M2e	50	4
V	M2e + VP6	50 + 10	4
VI	M2e + Al(OH)_3_	50 + 100	4
VII	Control	-	4

PP, P particle; VP6, VP6 nanosphere.
